# The Impact of the COVID-19 Pandemic on Informal Caregivers of People With Parkinson’s Disease Residing in the UK: A Qualitative Study

**DOI:** 10.1177/08919887221135555

**Published:** 2022-10-21

**Authors:** Daniel Rippon, Annette Hand, Lorelle Dismore, Roberta Caiazza

**Affiliations:** 1 5995 Northumbria University, Newcastle Upon Tyne, UK; 2 Northumbria Healthcare, 6072 NHS Foundation Trust, North Shields, UK

**Keywords:** informal caregiver, parkinson’s disease, Covid-19, caregiver burden

## Abstract

Informal caregivers can experience various demands when providing care and support for People with Parkinson’s disease (PwP) in their own homes. The outbreak of SARS-CoV-2 and public health strategies employed to mitigate the spread of COVID-19 have presented challenges to the general populace on a global basis. The present study used a qualitative research design to explore how the COVID-19 pandemic has impacted informal caregivers in their role of providing care for PwP in their own homes. A series of 1:1 semi-structured interviews were conducted with 11 informal caregivers of PwP (*M* age = 72.64 years, *SD* = 8.94 years). A thematic analysis indicated that 1) vulnerabilities to COVID-19, 2) home maintenance & activities of daily living and 3) engagement with healthcare services were 3 themes that provided indications on how the COVID-19 pandemic impacted informal caregivers of PwP. The present study provides illustrations of how being an informal caregiver of PwP and being identified as high risk to COVID-19 can present challenges to the process of caring for loved ones who are also vulnerable to SARS-CoV-2. The results of the present study highlights the necessity to develop strategies to ensure that informal caregivers have the necessary resources to provide care for PwP in their homes and also maintain their own well-being in the post COVID-19 era.

## Introduction

Informal caregivers are defined as unpaid carers who are often family members or friends that provide care for people who have a disability, mental health condition or chronic disease.^1^ In the UK, it has been estimated that informal caregivers save the state an estimated £132 billion per year through the provision of unpaid care.^2^ It has also been reported that the number of informal caregivers in the UK increased from an approximate 9.1 million to 13.6 million after the outbreak of SARS-CoV-2 in 2019.^3^ SARS-CoV-2 is an airborne virus that can cause either mild symptoms, such as a repetitive cough, to more severe symptomatology as failure of the respiratory system.^4^ In January 2020, the World Health Organisation announced a state of Public Health Emergency of International Concern in response to the global spread of SARS-CoV-2.^5^ In March 2020, the UK announced a public health strategy that included social and physical distancing measures to mitigate the spread of SARS-CoV-2.^6^ These measures included the instruction to only leave the house to 1) shop for essentials, 2) to engage with an outdoor exercise session for a 1-hour period on an individual basis or with members of the household, 3) to receive essential medical care and 4) travel for work purposes in situations where working from home was not possible.^7^. People with Parkinson’s disease (PwP) who resided in their own homes were observed to be vulnerable to experiencing an exacerbation or worsening of symptoms during periods of stay at home orders that aimed to mitigate the spread of COVID-19.^8^ This would suggest that the care needs of PwP may have been impacted by the outbreak of SARS-CoV-2 and public health strategies employed to mitigate the spread of COVID-19. It has been acknowledged that informal caregivers have an essential role in meeting the care needs of PwP who reside in their own homes.^9^ Thus, it is necessary to gain an understanding of how the COVID-19 pandemic, and the public health strategies employed to mitigate the spread of SARS-CoV-2, have impacted informal caregivers of PwP.

The symptoms of Parkinson’s disease are categorised as 1) motor and 2) non-motor symptoms. The motor symptoms of Parkinson’s disease include bradykinesia, rigidity of muscles, resting tremor and postural instability.^10^ Non-motor symptoms of Parkinson’s disease include increased sensitivity to pain, orthostatic hypotension, depression, urinary dysfunction, constipation, cognitive impairment, psychosis and sleep disorder.^11^ PwP have been identified as a vulnerable group to SARS-CoV-2 as muscular rigidity within the respiratory system can impair the cough reflex, which can lead to severe negative health consequences in the event of contracting COVID-19.^12^ One of the hallmark symptoms of Parkinson’s disease is also cognitive inflexibility and difficulty in adapting to novel rules.^13^ The public health management of SARS-CoV-2 has introduced various novel behavioural changes at the societal level to mitigate the spread of infection, such as the communal use of face masks and keeping a physical distance of 2 metres from others within indoor settings.^14^ It has been argued that the requirement to adapt to novel situations and rules associated with public health strategies to reduce the spread of COVID-19 could elicit stressful situations for PwP due to cognitive inflexibility and difficulties in adapting to change.^15^ For example, a study conducted in Italy observed that PwP experienced significant reductions in physical exercise due to lockdown restrictions, which was associated with an increase in symptoms of depression and worsening of both motor and non-motor symptoms of Parkinson’s disease.^8^ PwP who were unable to engage with physiotherapy services due to stay at home orders were also observed to have increased vulnerability to falls, reduced mobility and degradation in gait ability.^16^ This is concerning given that regular engagement in exercise and physiotherapy can be a protective factor in ensuring the physical health needs and optimal mobility of PwP.^17^ The social restrictions attached to COVID-19 was also posited as a risk factor of inducing clinical depression and anxiety in PwP who have severe motor disorders.^18^ Furthermore, a study conducted in an outpatient clinic in Italy observed that PwP experienced lower mood and greater symptoms of depression after the outbreak of COVID-19^19^ while the process of being confined to the home setting has also been purported to contribute to sleep disturbances in PwP.^20^ Thus, previous research would suggest that the outbreak of COVID-19 and the restrictions attached to the pandemic have potentially impacted the biopsychosocial well-being of PwP. This is also concerning for the welfare of informal caregivers of PwP as greater symptom severity in care recipients has been associated with vulnerabilities to stress, depression and degradation in physical health within familial carers of PwP.^21^

Quantitative research designs have previously been employed to assess how the COVID-19 pandemic has impacted informal caregivers of PwP. For example, a survey study conducted in Iran indicated that informal caregivers of PwP reported higher levels of anxiety due to the COVID-19 pandemic in comparison to non-caregiver controls who were matched on age and gender.^22^ A study conducted in Italy indicated that the process of providing informal care for PwP who had more severe anxiety, cognitive impairment and urinary dysfunction during a 10-day lockdown period was associated with higher caregiver stress and burden within familial caregivers.^23^ Another study conducted in Italy observed that a 40 day lockdown period led to increased levels of burden experienced by informal caregivers when caring for PwP who had lower levels of autonomy in completing activities of daily living (ADLs), such as mobilising in the home, bathing and eating.^24^ This would suggest that some public health strategies concerning COVID-19, such as stay-at-home orders, may have impacted the levels of anxiety, burden and stress experienced by informal caregivers of PwP. However, a scoping review has indicated that there is a lack of studies using qualitative research designs to illustrate lived experiences of how the outbreak of SARS-CoV-2 has impacted informal caregivers of PwP^25^ Thus, applying the principles of an inductive thematic analysis would enable informal caregivers of PwP to use their own words to illustrate their lived experiences^26^ to provide explanations and highlight specific situations as to how the outbreak of SARS-CoV-2 may have affected the process of providing informal care for PwP.

The long-term provision of physical, social and emotional support for loved ones with Parkinson’s disease can potentially be challenging and have detrimental consequences on the well-being of informal carers, which can then impact the welfare of care recipients.^27^ The outbreak of SARS-CoV-2 has potentially presented further demands as it has affected the general population in various ways, such as worrying about the impact of the COVID-19 pandemic,^28^ inducing a sense of physical loneliness^29^ and financial stress.^30^ The present study aimed to use a qualitative research design to specifically explore the experiences of informal caregivers of PwP on how the COVID-19 global pandemic has impacted their role of caring for a relative with Parkinson’s disease in their own home.

## Method

### Research Approach

The present study used a qualitative research design in which 1:1 semi-structured telephone interviews were conducted to explore participants’ experiences of providing informal care for PwP during the COVID-19 pandemic. Author RC conducted the telephone interviews and transcribed the audio recordings verbatim. An inductive Thematic Analysis of the dataset was conducted^31^ to ascertain the commonalities in participants’ experiences of providing informal care to a PwP after and during the global outbreak of SARS-CoV-2.

### Participants

A purposive sample of 11 informal carers of PwP, who resided in the North-East of England, were recruited to take part in the present study. Participants’ age ranged from 52 and 84 years (*M* = 72.64 years, *SD* = 8.94 years). Participants were recruited via a larger longitudinal programme of research entitled ‘The Northumbria Care Needs Project’. Previous studies have been published from this programme of research that has illustrated the care needs of people with moderate to advanced Parkinson’s disease who reside in their own home^32^ and the role of their informal caregivers^9^ prior to the outbreak of Covid-19. Thus, all participants were informal caregivers of a person with a diagnosis of moderate to advanced Parkinson’s disease who were under the care co-ordination of an NHS community Parkinson’s service. Please refer to Table 1. for details of participants’ gender identity, relationship with care recipient and living situation.

**Table 1.** Participant demographic information.

Participant ID	Gender	Age	Relationship with Care Recipient	Living Situation
1	Male	74	Spouse	Living with care recipient
2	Male	84	Spouse	Living with care recipient
3	Female	71	Spouse	Living with care recipient
4	Female	68	Spouse	Living with care recipient
5	Male	77	Spouse	Living with care recipient
6	Female	75	Spouse	Living with care recipient
7	Male	79	Spouse	Living with care recipient
8	Female	67	Spouse	Living separately from the care recipient
9	Female	52	Daughter of care recipient	Living separately from the care recipient
10	Female	69	Spouse	Living with care recipient
11	Female	83	Spouse	Living with care recipient

### Materials

An interview schedule was developed with questions that aimed to guide participants to discuss their experiences of informal care provision during the SARS-CoV-2 pandemic. Details of the interview schedule can be found in Table 2.

**Table 2.** Details of the questions included in the interview schedule.

Interview Schedule
1. How has your role as a carer been impacted by the COVID-19 pandemic?
**2. What aspects of your lifestyle have been impacted by the COVID-19 pandemic?**
3. How have the restrictions associated with COVID-19, for example the lockdowns, impacted your ability to provide care effectively?
**4. What type of support have you been able to access, during the COVID-19 pandemic, to assist you in your role as a carer?**
5. What type of support have you been unable to access during the COVID-19 pandemic?
**6. What type of support do you think would assist you in your role as being a carer during this pandemic?**
7. Is there anything else you would like to say about your role of caring for someone with Parkinson’s during the COVID-19 pandemic?

### Statement of Ethical Approval

Ethical approval was granted by Newcastle North Tyneside Research Ethics Committee and local R&D approvals prior to data collection in a National Health Service (NHS) secondary care, Parkinson’s department.

### Procedure

The interviews were conducted between May - July 2021. To provide some context on the COVID-19 related restrictions in England during recruitment of participants and data collection, please note that during April 2021, people were not permitted to socialise with others from different households within indoor settings. In May 2021, indoor hospitality had re-opened to the public in which groups of up to 6 people could meet. Social distancing measures were removed in England on 19th July. Participants who agreed to take part in the study were contacted by author RC via telephone in order for the 1:1 semi-structured interviews to be conducted. Participants were provided with details of the aims of the semi-structured interviews and what participation in the present study would consist of. Participants were asked to provide verbal consent to document their informed consent to take part in this study. Once informed consent to take part had been obtained, participants were then informed that a Dictaphone would be started to audio record the interview. The researcher then commenced with asking participants the questions included in the interview schedule, as illustrated in Table 2. The duration of the interviews was approximately 30 minutes. Once an interview had been completed, participants were notified that the Dictaphone had been switched off.

### Procedure for Analysis

The audio recordings of interviews were transcribed verbatim, in which transcripts were anonymised. The 6 stages of thematic analysis^31^ were applied to the dataset. Author DR coded the transcripts in accordance with passages of the dataset that provided illustrations of how the COVID-19 pandemic had impacted informal carers of PwP. Once the codes had been collapsed into overarching themes, the initial thematic map was sent to authors AH, LD and RC for consideration and discussion. Once there was agreement that the thematic map provided an accurate representation of participants’ experience of providing informal care for PwP after the outbreak of SARS-CoV-2, as documented in the transcripts, the write-up of the thematic analysis commenced. The following section of this paper provides details of the reported thematic analysis for the present study.

## Results and Discussion

**Figure 1.**
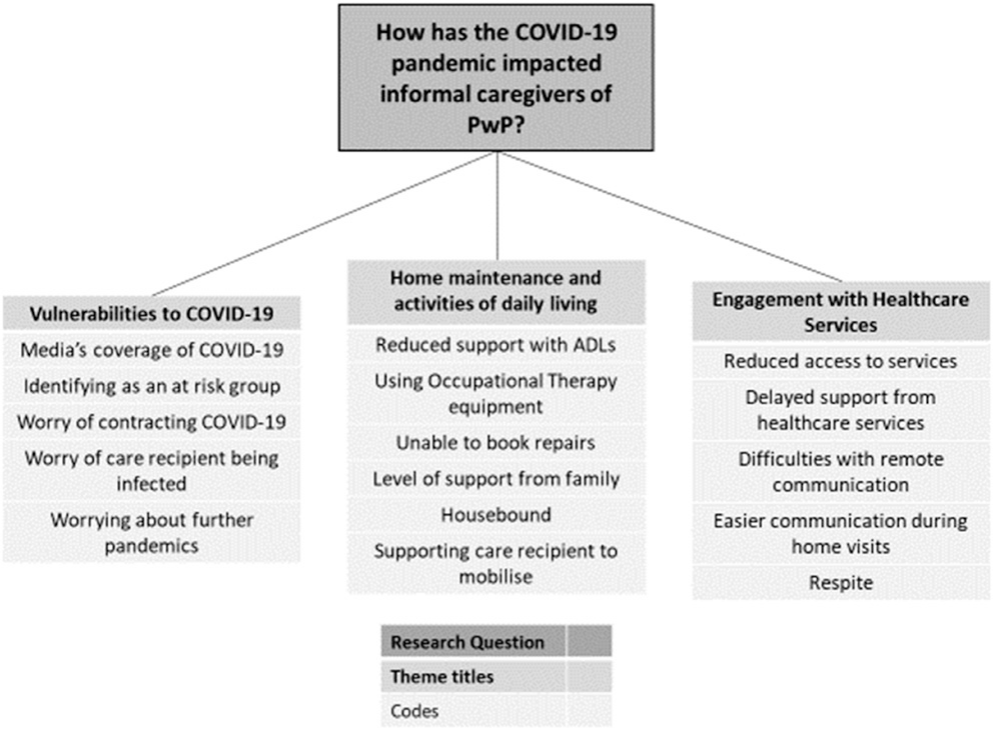
A thematic map of the factors that impacted informal caregivers of PwP after the onset of the SARS-CoV-2 pandemic.

A thematic analysis of the dataset indicated 3 key themes that illustrated how the COVID-19 pandemic had impacted informal caregivers of PwP, which were 1) vulnerabilities to SARS-Cov-2, 2) home maintenance and activities of daily living and 3) engagement with healthcare services. Figure 1. provides an illustration of how codes were incorporated into overarching themes. This section will provide extracts from the dataset, which will be discussed in accordance with relevant literature to demonstrate how the outbreak of SARS CoV-2 influenced informal caregivers of PwP.^33-43^

## Vulnerabilities to COVID-19

Participants stated the difficulties of providing informal care for loved ones with Parkinson’s disease whilst acknowledging their own vulnerabilities to the symptoms of COVID-19. Participants discussed how engaging with media coverage and national briefings related to COVID-19 could have negative consequences on the mood of informal caregivers.

“It got to the point where we stopped looking at the news with Boris’ [Prime Minister of the UK at the time of data collection] updates as it was always about people dying, which was quite depressing. You know, my wife [care recipient] and I got upset about it. Although that is a fact with the virus, we didn’t want to know all those horrible numbers and everything else”.

(Participant 5)

It has been posited that regular viewing and the content of COVID-19 related news updates could have had detrimental consequences on well-being within the general population.^44^ For example, it has been argued that the content of governmental press briefings could be perceived as comprising of overestimations on how well resourced the UK was in managing the COVID-19 pandemic and regular amendments to public health strategies, all of which could have caused confusion and uncertainties in the general populace.^45^ The quote above suggests that informal caregivers of PwP were a particular subgroup of the UK population who found that engaging with media and governmental announcements on COVID-19 had a detrimental impact on mental well-being. This degradation in mood could have been due to informal caregivers, along with their care recipients, being identified and reported as being a high-risk group to the symptoms of COVID-19 due to being an older person and having underlying health conditions.

“We were very concerned as it was always our age bracket of being 70 to 80 with underlying health conditions that were the most vulnerable. When you are ill, it is not a very nice prospect”.

(Participant 5)

The present study indicated that contracting COVID-19 was of a serious concern for informal caregivers who had physical health issues of their own. This is concerning as it has been acknowledged that older informal caregivers of PwP may also be vulnerable to physical ailments, which can impact perceived quality of life (Morley et al., 2012). Furthermore, it has been observed that PwP may also be particularly vulnerable to the symptoms of COVID-19 as SARS CoV-2 can potentially accelerate aging of the brain and also exacerbate both motor and non-motor symptoms of Parkinson’s disease.^46^ Given that PwP were identified as a high-risk group from COVID-19, the quote below illustrates how informal caregivers were concerned about contracting SARS-CoV-2 in the community and then infecting their care recipient.

“*We were quite frightened to go out, until we had both been double jabbed [vaccinated against COVID-19]. We have been concerned, especially with the Parkinson’s as we didn’t want to expose my husband [care recipient]…I have made sure it has been me that picks the meds up. We made the decision that I would collect this with my mask. We were very strict with the restrictions and ensured we followed what we were supposed to do”.*

(Participant 10)

The quote above indicates how informal caregivers of PwP could potentially put themselves at risk when required to leave the household to go out into community settings to collect essential resources, such as medication, for their care recipient. In March 2020, shopping for essentials items and obtaining medical care were some of the few communal activities that were permitted during a time where stay at home orders were mandated in the UK to mitigate the spread of SARS-CoV-2.^7^ Between September 2020 and March 2021, it was posited that attending communal settings, such as hospitals, could increase the risk of contracting and then contaminating a given household with SARS-CoV-2.^47^ Thus, the present study indicated that even with adherence to public health strategies, such as the communal use of face masks, some informal carers had the demand and risks associated with going out into community settings to collect essential medical resources to ensure the on-going treatment of care recipients. The quote below also suggested that some participants were worried about the potential for future pandemics and how further restrictions could perpetuate a sense of loneliness within informal carers and concerns on how to meet the needs of care recipients.

“I think during a pandemic and considering the number we have had in the 21^st^ century compared to the 20th century, the number [of pandemics] are likely to pop up again over the next 5 or 6 years where we are going to have another one at least. That means more isolation of the carer. It is also means the isolation of the person who has the problem [Parkinson’s disease]”.

(Participant 3)

The quote above illustrates how worrying about the potential of future pandemics and notions of being isolated could be a concern for informal caregivers of PwP. The COVID-19 pandemic has shown to potentially induce acute panic, anxiety and post-traumatic stress in the general populace and it has been argued that mental health services need to be set up to support people through potential future pandemics.^48^ It could be that informal caregivers of PwP may also benefit from specialised support services that aim to reduce vulnerabilities and support familial carers in meeting the needs of care recipients within their own homes in the post COVID-19 era.

## Home Maintenance and Activities of Daily Living

Participants illustrated some of the difficulties that occurred during periods of lockdown in terms of completing home maintenance duties and supporting care recipients to engage in activities of daily living (ADLs) on an independent basis. ADLs comprise of basic and instrumental tasks.^49^ Basic ADLs include 1) mobilising from one position to another, 2) feeding, 3) dressing, 4) personal hygiene, 5) continence and 6) toileting. Instrumental ADLs include 1) transportation & shopping, 2) managing finances, 3) meal preparation, 4) house cleaning, 5) managing communication with others and 6) managing medications. Participants stated that periods of lockdown meant that professionals, such as cleaners and engineers, were unable to attend their home setting to assist with the household duties and repairing of essential appliances.

“Not having people around who normally help. So, we normally have a cleaner and a lady who helps with the ironing. But neither of these came in as we were shut in…I couldn’t easily ring someone as an electrician as you couldn’t have people at home. I think those things dwell on the mind”.

(Participant 3)

A previous meta-analysis has suggested that providing care for PwP who have greater dependency with ADLs is associated with higher distress and burden in informal caregivers.^50^ The quote above illustrates how some of the protective factors that could be used to support informal caregivers with ADLs, such as accessing professional cleaning and electrician services, were not available during periods of stay-at-home orders. The quote below also illustrates how stay at home orders inhibited informal caregivers from accessing support from family members to assist with ADLs, such as shopping.

“I haven’t been able to get the people we need to come in either. It’s made things very difficult with all the restrictions. I haven’t been able to have my family to come and help me with my husband [care recipient] when I needed to go and do the shopping and things like that”.

(Participant 6)

It has been posited that a lack of support from other family members can lead to carers of PwP to feel socially isolated, abandoned and unsupported in their role of informal caregiver.^51^ The views expressed by participants in the present study suggested that the COVID-19 pandemic enforced a situation where family members, who resided externally to the household, were unable to provide essential support to informal caregivers of PwP. However, other participants illustrated how the availability of family members, who were able to access their homes during periods of lockdown, was integral to the completion of essential household maintenance.

“We have spotlight in the bathroom. One of them started to go before the pandemic so we knew they were going to go and during lockdown they did. We don’t have a window in the bathroom. I am not great on my feet and my [Husband] shouldn’t be up there [trying to change light bulbs]. Fortunately, my son in law was able to sort that. But say I didn’t have the son in law, then slowly over the time the lights would have stopped working”.

(Participant 3)

It has been argued that the support of relatives can be crucial in providing informal caregivers with the stability and resources required to meet the care needs of PwP.^52^ During the peak lockdown measures in the UK, which occurred between March and May 2020, public health guidance indicated that single adult carers could join a social bubble with another household to ensure that the needs of people who required continuous care could be met.^53^ The quote above illustrates how the ability to join a social bubble with other family members was essential to informal caregivers in ensuring the upkeep of essential household maintenance during periods of lockdown.

However, the quote below suggested that informal caregivers could encounter difficulties when attempting to support their care recipient to use mobility aids and mobilise around the household without direct input of healthcare professionals, such as occupational therapists and physiotherapists.

“*There is one item of equipment that we use to transfer my wife from the landing to the bed and the toilet, which is like a walker. My wife stands on it and it has 2 larger wheels and 4 smaller wheels that work on the carpet. That does knock it out of us a little bit, I’m just about exhausted after using it”.*

(Participant 2)

Previous research has indicated that for PwP who reside in their own home, 80% of falls occur within the household setting.^54^ Postural instability and gait difficulties have been cited as symptoms of Parkinson’s that can increase the risk of falls in the home setting.^55^ Bedroom and bathrooms have been identified as 2 particular areas in the household that can present challenges for PwP when mobilising, which can potentially lead to freezing of gait and falls.^56^ The quote above suggested that during periods of lockdown, informal caregivers could experience exhaustion due to the demand of independently supporting care recipients to use occupational therapy equipment to ensure safe mobilisation and transfer between areas of the household, such as the bedroom and bathroom areas. Thus, it could be that not having access to home support from occupational therapy services placed further demands on informal caregivers during periods of lockdown, such as the requirement to mobilise care recipients around the household.

Participants stated that there were notable reductions in the well-being of their care recipient during times when it was not possible to have face-to- face contact with family, friends, and healthcare professionals.

“It has certainty curtailed many visits and outdoor activities and that sort of thing, just to get my wife [care recipient] out, as she spends most of her time indoors. Not seeing the doctors and friends and family and that sort of thing, which would help my wife’s wellbeing, has been a big drawback”.

(Participant 5)

Although stay at home orders in the UK were deemed necessary to mitigate the spread of SARS-CoV-2, public health concerns were raised on how reductions in face-to-face contact with others could lead to loneliness, which can have negative consequences on physical and mental well-being.^57^ Participants suggested that the requirement to stay at home induced a sense of loneliness and reduced the mood within care recipients, which was worrying for informal caregivers of PwP.

*“She spends a lot more time at home now, which is a worry for us as she has deteriorated… my mam suffered terribly with loneliness and depression. She was very very depressed which was quite hard for me to support her”.*

(Participant 9)

The quote above is consistent with previous research in which periods of lockdown were associated with symptoms of anxiety, intrusive thoughts about the pandemic, anger and irritability within PwP, which could then place greater burden on informal caregivers^24^

The quote below also illustrates how informal caregivers were required to encourage and reinforce the mobility of care recipients to negate PwP from experiencing physical deterioration due to the requirement to spend more time inside of the household.

“He [care recipient] slowed down his movement. He has become more hesitant and sticks more in doorways etc. So, we have had to think about and remind him to do things, such as step through [the doorways in the house]. I’ve also had to remind him more about his tablets than I had to do before”.

(Participant 9)

Sedentary lifestyles that comprise of staying indoors and being inactive for prolonged periods of time have been associated with deficits in mobility and cognitive functioning within PwP.^58^ The present study suggested that a consequence of stay-at-home orders was that PwP may develop sedentary lifestyles during periods of lockdown, which led to reduced mobility and memory functioning. This placed greater demands on informal carers in ensuring that care recipients were provided with prompts to encourage mobilising in the home and completion of essential tasks, such as taking medications as prescribed. Participants also illustrated how informal caregivers were proactive in using the time permitted to go outdoors in order to support care recipients to engage in exercise to further negate degradation in mobility.

“Exercise has been the best for us, we have been out every day either walking or on the bike. He (husband) finds that if he doesn’t get out daily, he seizes up. He’s always been a very fit and active man. It does us both good”.

(Participant 10)

On 23rd March 2020 in the UK, it was announced that people were permitted to leave the house once per day in order to engage with an outdoor exercise session, either on an independent basis or with other members of the household^7^**.** The results of the present study suggested that the onus was placed on informal caregivers of PwP to use the time outdoors in a way that encouraged care recipients to engage in exercise in order to offset sedentary lifestyles during periods of stay-at-home orders. The support of informal caregivers in mobilising care recipients during periods of lockdown could be deemed essential, given that exercise can be beneficial for motor functioning in terms of gait, balance and strength, and non-motor functions, such as reducing depressive symptoms, apathy and fatigue in PwP.^59^ Furthermore, the results of the present study also suggested that participating in exercise alongside care recipients may yield beneficial outcomes for informal caregivers of PwP.

## Engagement with Healthcare Services

Participants discussed how the public health measures employed to mitigate the spread of COVID-19 impacted the way they accessed healthcare services. Participants stated that the requirement to reduce interactions in the community meant that they relied on healthcare services to deliver home treatment for care recipients. However, participants experienced delays in the provision of home treatment, which then delayed care recipients from receiving essential care from healthcare professionals, such as general practitioners and physiotherapists.

“We haven’t been able to get the help or cover that we have needed from a doctor. My husband [care recipient] hasn’t seen a doctor at home for as long as I can remember. Some of the things and facilities, for example physiotherapy, we went a long time before anyone was able to come here”.

(Participant 6)

The consistent provision of healthcare services in the homes of PwP has been identified as being an essential component of ensuring that the bespoke needs of care recipients are met.^60^ It has been argued that disruption in medical care can elicit distress and increase demands placed on informal carers of PwP.^27^ The quote above illustrates how restrictions employed to mitigate the spread of SARS-CoV-2 potentially interrupted and delayed access to healthcare services that were essential to the welfare of care recipients. This is concerning given that interruptions in care delivery can potentially exacerbate pain, rigidity and tremors within PwP.^61^ However, the quote below indicates that some participants were mindful that delays in healthcare provision was due to frontline healthcare staff also being affected by the COVID-19 pandemic.

“The situation now has changed for the PD [Parkinson’s Disease] support team. I think that it’s like everything else. It’s dealing with someone that is remote. You can speak to the PD team, but you can’t speak to the consultant. The [Parkinson’s Disease] team are emailing them and then you would get an email back, a second-hand report of what the consultant had said. So, I suppose that was emphasised by COVID-19 as you know all the personnel problems they had. For example, shortage of staff, infected or isolating, that sort of thing”.

(Participant 5)

The COVID-19 pandemic presented various challenges for frontline healthcare services, such as staff shortages, constraints on medical resources, supply issues for personal protective equipment (PPE) and preparing for potential subsequent waves of SARS-CoV-2.^62^ Frontline healthcare staff, who worked in settings such as care homes, reported to have been vulnerable to psychological distress during the COVID-19 pandemic due to staff shortages, fear of being infected, worrying about infecting others and loss of residents/colleagues to SARS-CoV-2.^63^ Furthermore, there were more reported incidences of healthcare professionals testing positive for COVID-19, which required periods of self-isolation, in comparison to the general population.^64^ The quote above illustrates how healthcare services dedicated to the on-going care of PwP could be depleted through members of staff testing positive for COVID-19 and self-isolating, which meant that informal caregivers experienced difficulties in accessing formal support at times of need. Participants also stated that the requirement to engage with healthcare services on a remote basis, rather than attend hospital settings and interact with staff on a face-to-face basis, presented barriers to having effective and timely communication with clinicians.

“It has limited us to go and see the consultant and the nurses face to face you know, which is obviously not the best way to try and describe some sort of medical condition…It was difficult to actually see a GP. They were very good about having a chat on the phone but to actually get into the surgery, these things were difficult to describe over the phone”.

(Participant 5)

Previous research that focussed on informal caregivers of people with a cancer diagnosis has suggested that telecommunications with healthcare professionals can be sufficient in obtaining professional support and guidance on caregiver duties.^65^ However, the quote above suggested that informal caregivers of PwP experienced difficulties in conveying the status and symptoms of their care recipient when required to communicate with healthcare professionals on a remote basis. Furthermore, participants stated that home visits from healthcare professionals during periods of lockdown, which enabled face-to-face treatment of care recipients, was more effective than engaging in remote telecommunications with clinicians.

“He was so isolated, especially when we were contacting people on the phone. It is different from how they talk to you and when they actually see you. So, I could explain to the nurse the difficulties, but it wasn’t until she actually saw us that she realised what we meant. I think with Parkinson’s, you can describe how you’re feeling, and you can describe something. But you can understand more when you see how someone actually does something”.

(Participant 3)

It has been recognised that PwP can be vulnerable to being housebound, which can inhibit engagement with healthcare services in clinical settings.^66^ The provision of homecare services as delivered by healthcare professionals, such as neurologists, social workers and nurses, can be essential in ensuring that PwP receive person centred care within their own homes.^66^ The quote below suggests that home visits from clinicians, such as physiotherapists, went someway to offsetting the detrimental effects of the pandemic on the mobility of PwP.

“We did have the physiotherapist. He has got in contact, which is good. He has also been to visit and we have been able to visit afterwards to keep up with his mobility… I think that is because his condition worsened during the pandemic. They [physiotherapists] helped in the sense that they gave the cues to keep reminding him to move until we could get out more and adapt to what we were doing”.

(Participant 3)

### General Discussion

The present study has indicated that since the outbreak of SARS-CoV-2 in 2019, the role of informal caregivers of PwP has been affected in various ways. Firstly, it must be acknowledged that informal carers of PwP who are of older age and have underlying health concerns are also vulnerable to the symptoms of SARS-CoV-2. At the time of writing, the public health strategies that curtailed face-to-face interactions within community indoor settings are no longer mandated in the UK. Yet, in March 2022, COVID-19 related hospitalisations have increased from 14.07 per 100,000 people up to 17.89 per 100,000 people within the UK (Office of National Statistics, 2022). This suggests that there is an on-going risk of communal transmission of SARS-CoV-2. The present study indicated that informal caregivers can potentially place themselves at risk when engaging in any caregiver duty that requires face-to-face interactions in community settings, such as collecting medication for a care recipient and shopping for groceries. It could be argued that items, such as medication and groceries, can be delivered to the homes of informal caregivers and PwP. However, as the present study has revealed, informal caregivers have potentially found the process of reducing social interactions and decreasing time spent out in community settings to have a deleterious effect on their well-being. It could be that strategies, such as the role out of vaccination programmes, could instil a sense of confidence within informal caregivers to re-engage with communal settings after going through periods of lockdowns and stay at home orders. For example, 2 doses of the Pfizer-BioNTech BNT162b2 vaccine has shown to provide older adults, aged 70 years of age and older, with protection from severe symptoms of SARS-CoV-2.^67^ An initial dose of Oxford–AstraZeneca ChAdOx1 nCoV-19, followed by a booster 12-weeks later, has also shown to provide protection from severe symptoms of COVID-19.^68^ However, other public health strategies, such as the communal use of face masks, have eased and are not mandated in some settings. Thus, there is a need to conduct further research to ascertain the extent to which informal caregivers have confidence in re-engaging with their communities and face-to-face interactions with others in the post COVID-19 era. It would also be beneficial in ascertaining as to whether particular strategies, such as compliance with vaccine programmes and the voluntary communal use of face masks, are associated with informal caregivers’ confidence and facilitation to re-engage with communal settings.

Family members were also observed to be an important source of support in assisting informal caregivers to complete essential household maintenance during periods of lockdown. The present study illustrated how household maintenance, such as changing light bulbs, can potentially place older informal caregivers in precarious situations in the home. Thus, having family members available to complete household tasks was beneficial in ensuring that informal caregivers and their care recipients were not putting themselves at risk of harm within their own home. However, the present study also revealed how reduced interactions with family members was particularly difficult for some informal caregivers of PwP during periods of lockdown. This raises an issue for those informal caregivers of PwP who do not have access to support from other family members in the post COVID-19 era. It has been argued that informal caregivers of PwP, who do not have access to a cohesive support network from relatives, can experience reductions in mental well-being and higher levels of carer burden.^69^ Furthermore, chronic carer stress and burden can potentially lead to PwP from leaving the residency of their own home into care home placement.^70^ This is concerning, given that care home placement can diverge from the wishes of informal caregivers of continuing to provide care for loved ones with Parkinson’s disease in their own homes.^71^ Although the present study focussed on the experience of providing informal care to PwP during the global COVID-19 pandemic, the results indicate that the absence of family support may increase demands on informal caregivers in terms of ensuring effective household maintenance, caregiving and supporting PwP with ADLs in their own home.

The present study also indicated that face-to-face interactions with healthcare professionals was more beneficial to informal caregivers, in ensuring that the care needs of PwP were being met, in comparison to having remote telecommunications with clinicians. It was suggested that informal caregivers may have difficulties in verbally communicating care recipients’ symptoms to clinicians via telecommunications. Previous research has shown that utilising remote methods of communication, such as videoconferencing, can also inhibit clinicians from providing comprehensive assessments of PwP’s motor and non-motor symptoms.^72^ The reason for clinicians missing assessment items during remote communications can be due to PwP’s inability to position themselves in front of a camera that allows assessment of the whole-body movement and technical difficulties caused by internet connection. Given that face-to-face interactions with care recipients may facilitate clinicians to complete comprehensive assessments of PwP, the use of telecommunications between informal caregivers and healthcare professionals may not have facilitated accurate clinical examinations during periods of lockdown. However, it has been noted that informal caregivers of PwP can be vulnerable to being housebound in situations where the symptoms of care recipients worsen and demands placed upon the carer increase.^73^ Thus, at a time where COVID-19 related restrictions have eased, there may still be a need to ensure that informal caregivers have the resources to arrange for care recipients to engage with home healthcare on a face-to-face basis with clinicians.

Some potential limitations of the present study require consideration when interpreting the results. Firstly, it must be noted that the 1:1 semi-structured interviews were conducted via telephone with participants. This method of data collection was used in order to avoid the requirement for face-to-face interactions during interviews to ensure participants’ and researcher safety at a time where communal transmission of COVID-19 was still a risk. Although telephone interviews can be beneficial in ensuring participant safety, the use of telephones may be problematic for participants who have mobility or other physical health issues that inhibit the operating of a device for prolonged periods of time.^74^ It must also be acknowledged that all participants who took part in this study were accessing healthcare services in the North-East of England. It has been purported that there is an inequity in the quality of community healthcare provision and homecare delivery of specialist treatments for PwP in the UK.^75^ Thus, informal caregivers of PwP who reside in other parts of the globe may have different experiences and perspectives on the engagement with healthcare services during periods of stay-at-home orders as those reported by the participants who took part in the present study.

Nonetheless, the present study has provided insights on some of the factors that require consideration to ensure that informal caregivers have the means to provide effective care for PwP within their own homes in this post Covid-19 era. More specifically, the results of present study suggests that informal caregivers of PwP may require support in facilitating care recipients with ADLs in their own home and to access healthcare services when required in their respective communities. This type of community support may be necessary for informal caregivers who still have concerns regarding the communal spread of SARS-CoV-2 in this post Covid-19 era. A potential strategy to promote effective care in the community for PwP is to ensure that informal caregivers have the resources to provide effective support for care recipients in their own home.^9^ Ensuring that informal caregivers have the resources to provide effective care can also negate premature care home placements for PwP.^76^ It has also been argued that the needs of informal caregivers require consideration when developing community support plans for PwP in order to negate caregiver burnout.^77^ Thus, an aim for the authors of the present study is to use the reported results to develop a community intervention that supports informal caregivers in their role of caring for loved ones with Parkinson’s disease in their own homes in terms of facilitating PwP with ADLs and their engagement with healthcare professionals in community settings. To further support informal caregivers in their role, the research team is also looking to develop a skills and training packages that aims to prevent and/or decrease mental and physical health problems associated with caregiving and to improve the quality of life of those caring for people with Parkinson’s disease.

In summary, the present study aimed to illustrate the lived experiences of informal caregivers on their role of providing care for PwP after the outbreak of SARS-CoV-2. The present study has revealed that informal caregivers recognise the negative consequences of contracting COVID-19 in terms of their own well-being and potentially infecting loved ones who have Parkinson’s disease. The worry of contracting COVID-19 in the community and bringing SARS-CoV-2 into their own home can have negative consequences on both the physical and psychological well-being of informal caregivers of PwP. Public health strategies that comprised of stay at home orders potentially reduced the availability of face-to-face support from relatives and healthcare professionals. It is essential that the lived experiences of informal caregivers inform the way in which social support and healthcare provision can be operationalised to ensure that familial carers have the resources to provide effective care for PwP within their homes in the post COVID-19 era.
